# 00005 Urine tumor DNA detects minimal residual disease in muscle-invasive bladder cancer treated with curative-intent radical cystectomy

**DOI:** 10.1017/cts.2021.690

**Published:** 2021-03-30

**Authors:** Kevin Chen, Pradeep S. Chauhan, Ramandeep K. Babbra, Wenjia Feng, Eric H. Kim, Zachary L. Smith, Vivek K. Arora, Aadel A. Chaudhuri

**Affiliations:** Washington University School of Medicine in St. Louis

## Abstract

ABSTRACT IMPACT: Urine tumor DNA non-invasively detects minimal residual disease and infers tumor mutational burden in locally advanced bladder cancer prior to radical cystectomy, which may potentially enable the selection of patients for bladder-sparing treatment or facilitate personalized adjuvant immunotherapy. OBJECTIVES/GOALS: Standard-of-care treatment for muscle-invasive bladder cancer (MIBC) is radical cystectomy. The inability to assess minimal residual disease (MRD) non-invasively limits our ability to offer bladder-sparing treatment. We sought to develop a liquid biopsy solution via urine tumor DNA (utDNA) analysis. METHODS/STUDY POPULATION: We applied uCAPP-Seq, a targeted sequencing method for detecting utDNA, to urine cell-free DNA samples acquired on the day of radical cystectomy from 42 patients with bladder cancer. utDNA variant-calling was performed non-invasively without prior tumor mutational knowledge. The overall utDNA level for each patient was represented by the non-silent mutation with the highest variant allele fraction after removing germline variants. Urine was similarly analyzed from 15 healthy adults. Tumor mutational burden (TMB) was inferred from the number of non-silent mutations detected in urine cell-free DNA by applying a linear relationship derived from TCGA whole exome sequencing of 409 MIBC tumors. RESULTS/ANTICIPATED RESULTS: utDNA levels were significantly higher in patients with residual disease detected in their surgical pathology compared to those who achieved a pathologic complete response (P = 0.002). Using an optimal utDNA threshold to define MRD detection, positive utDNA MRD significantly predicted the absence of pathologic complete response with a sensitivity of 81% and specificity of 81%. Positive utDNA MRD also portended significantly worse progression-free survival (HR = 7.4; P = 0.03) compared to negative utDNA MRD. Furthermore, we applied a linear relationship (Pearson r = 0.84; P < 0.0001) to identify patients with high inferred TMB who may have been candidates for early immune checkpoint blockade. DISCUSSION/SIGNIFICANCE OF FINDINGS: utDNA MRD analysis prior to surgery correlated significantly with pathologic response and progression-free survival, which may help select patients for bladder-sparing treatment. utDNA can also non-invasively infer TMB, which could facilitate personalized adjuvant therapy for patients in the future.

